# Circular RNA circFCHO2(hsa_circ_0002490) promotes the proliferation of melanoma by directly binding to DND1

**DOI:** 10.1007/s10565-024-09851-y

**Published:** 2024-02-05

**Authors:** Yang Yang, Jianrui Li, Chuanyuan Wei, Lu Wang, Zixu Gao, Kangjie Shen, Yinlam Li, Ming Ren, Yu Zhu, Yiteng Ding, Chenlu Wei, Tianyi Zhang, Shaoluan Zheng, Nanhang Lu, Jianying Gu

**Affiliations:** 1 Department of Plastic and Reconstructive Surgery, Zhongshan Hospital, Fudan University, No. 180 Feng Lin Road, Shanghai, 200032 China; 2 Department of Plastic and Reconstructive Surgery, Zhongshan Hospital (Xiamen), Fudan University, Xiamen Clinical Research Center for Cancer Therapy, Xiamen, 361015 China

**Keywords:** Melanoma, circFCHO2, DND1, PI3K/AKT, RNA-binding protein

## Abstract

Circular RNAs (circRNAs) have been documented to play crucial roles in the biology of various cancers. However, their investigation in melanoma is still at an early stage, particularly as a broader mechanism beyond acting as miRNA sponges needs to be explored. We report here that circFCHO2(hsa_circ_0002490), a circRNA encompassing exons 19 and 20 of the FCHO2 gene, exhibited a consistent overexpression in melanoma tissues. Furthermore, elevated circFCHO2 levels demonstrated a positive correlation with the malignant phenotype and poor prognosis among the 158 melanoma patients studied. Besides, we observed that heightened levels of circFCHO2 promoted melanoma cell proliferation, migration, and invasion in vitro, along with contributing to tumor growth in vivo. Furthermore, we found differences in the secondary structure of circFCHO2 compared to most other circular RNA structures. It has fewer miRNA binding sites, while it has more RNA binding protein binding sites. We therefore speculate that circFCHO2 may have a function of interacting with RNA binding proteins. Mechanistically, it was confirmed by fluorescence in situ hybridization (FISH), RNA-pull down, RNA immunoprecipitation (RIP), and western blotting assays that circFCHO2 interacts with dead end protein homolog 1 (DND1) and reverses the inhibition of the PI3K/AKT signaling pathway by binding to DND1. Our findings reveal that circFCHO2 drives melanoma progression by regulating the PI3K/AKT signaling pathway through direct binding to DND1 and may serve as a potential diagnostic biomarker and therapeutic target for the treatment of melanoma.

**Graphical Abstract:**

Headlights: CircFCHO2 is highly expressed in melanoma. High circFCHO2 levels were positively correlated with poor prognosis in 158 melanoma patients. CircFCHO2 is involved in the regulation of the PI3K/AKT signalling pathway by binding to DND1. CircFCHO2 could serve as a potential biomarker and therapeutic target for the management of melanoma. 
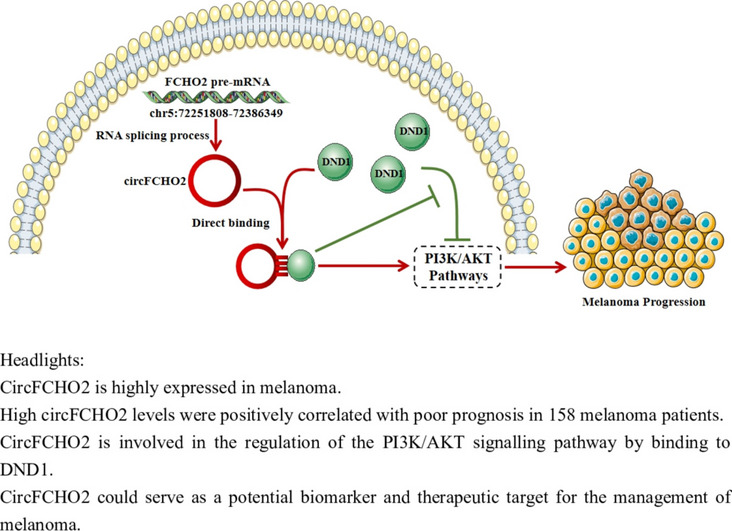

**Supplementary Information:**

The online version contains supplementary material available at 10.1007/s10565-024-09851-y.

## Introduction

Melanoma is a malignant tumor that originates from melanocytes, is prone to metastasis and recurrence, and has a very high mortality rate (Guo et al. 2015). Currently, the annual number of new cases in China is approximately 20,000 (Si et al. 2020), and in recent years it has continued to increase with an annual growth rate of 3–5% (Siegel and Miller 2023). Due to a lack of awareness about melanoma, many patients are diagnosed in the intermediate to advanced stages when seeking medical attention, missing the optimal window for treatment. Advanced melanoma poses significant challenges for surgical intervention and is associated with a bleak prognosis. The overall 5-year survival rate stands at a mere 4–10% (Long et al. 2015). Traditional adjuvant treatment methods are limited and cannot improve overall survival, with significant side effects (Dummer et al. 2020). In December 2019 and March 2020, respectively, dabrafenib and trametinib were sequentially approved for the treatment of B-Raf mutation, characterized by higher malignancy and a poorer prognosis. These approvals included indications for both first-line advanced and adjuvant treatment in China. This marked a significant milestone as it became the inaugural and sole targeted treatment regimen with dual applications for advanced and adjuvant treatment within the realm of melanoma in China (Association , 2021). However, the B-Raf mutation rate in Chinese patients is only about 25%, and some patients are prone to drug resistance in a short period of time, resulting in a low overall response rate of targeted therapy (Weber et al. 2017). Therefore, it is still important for the clinical diagnosis and treatment of melanoma to explore and improve the detailed mechanism of melanoma progression, further find new tumor intervention targets, and analyze new molecular mechanisms.

In recent years, a large number of non-coding RNAs, especially circular RNAs, have been reported to play a crucial role in the regulation of biological functions in various tumors, which has attracted increasing attention. At present, studies have found that circular RNA is implicated in the onset and progression of liver cancer, gastric cancer, esophageal cancer, colorectal cancer, breast cancer, prostate cancer, melanoma, basal cell carcinoma, and other malignant tumors (Hsiao et al. 2017; Chen et al. 2018, 2017; Yang et al. 2017, 2019; Qiu et al. 2018; Zeng et al. 2018; Wang et al. 2018; Zhong et al. 2017; Abdelmohsen et al. 2017), which has great application potential in the clinical diagnosis and treatment of various types of tumors. Circular RNA was discovered as early as the 1970s (Diener 1971; Hsu and Coca-Prados 1979), but researchers at that time initially regarded it as a byproduct of non-functional RNA molecules formed by “erroneous” splicing, which did not receive much attention, but with the ongoing advancement of bioinformatics and RNA sequencing technology, circular RNA has been found to be widely distributed in various eukaryotic histiocytes (Liu and Chen 2022), and its structure and rich biological functions have also been progressively unveiled. The main difference between circular RNA and linear RNA is that they do not have a cap structure at the 5′ end and a polyadenylate tail structure at the 3′ end. Instead, they are covalently linked from head to tail to form a closed ring structure, making them highly stable (Liu and Chen 2022; Xiang Liu and Zhang 2022). Due to their highly conserved and stable properties, the expression levels of circular RNAs are even higher than the corresponding linear RNAs in certain specific tissues and developmental stages. Numerous studies have also confirmed that their specific expression can be considered an important molecular marker for diagnosis and potential intervention and treatment targets in various tumor and other disease processes (Hsiao et al. 2017; Chen et al. 2018, 2017; Yang et al. 2017, 2019; Qiu et al. 2018; Zeng et al. 2018; Wang et al. 2018; Zhong et al. 2017; Abdelmohsen et al. 2017; Liu and Chen 2022). Therefore, basic research based on circular RNA has significant clinical translational importance.

In recent years, many studies based on circular RNA have focused on the mechanism of action as a ceRNA. As a direct effector of almost all important biological functional activities, the mode of interaction between proteins and circular RNA has not been fully elucidated. And according to the secondary structure analysis of some circular RNAs, one type of circular RNA can simultaneously act as a miRNA “sponge” and a protein interaction factor (Meng et al. 2022). The progressively evolving research on the interaction patterns between circular RNA and proteins has provided us with fresh insights into its biological significance. However, research on the interaction between circular RNA and proteins in melanoma is still very scarce, and its specific mechanism awaits our further exploration and improvement.

In this study, we identified a pro-tumorigenic circular RNA, circFCHO2, by RNA sequencing and quantitative real‑time PCR (qRT-PCR). We found a significant upregulation of circFCHO2 in melanoma tissue, and its promotion of melanoma progression was evident both in vivo and in vitro. Subsequent investigations have revealed that circFCHO2 might be implicated in the regulation of the PI3K/AKT pathway through its direct binding to DND1. Collectively, our findings suggest a crucial role for circFCHO2 in melanoma progression. This circular RNA holds promise as a potential biomarker and therapeutic target for melanoma.

## Methods

### Patients and samples

A total of 158 melanoma cases, along with corresponding normal tissues, and 9 pigmented nevus tissues were randomly sourced from the Department of Plastic and Reconstructive Surgery, Zhongshan Hospital, Fudan University (Shanghai, China). All specimens were securely preserved at − 80℃. Each patient underwent comprehensive total resection, followed by a meticulous histological and pathological examination conducted by two pathologists. None of the patients received any form of radiotherapy or chemotherapy before undergoing surgery, and all participants were provided with detailed clinical, pathological, and follow-up data. This study received approval from the Ethics Committee of Zhongshan Hospital, Fudan University, and each patient willingly provided written informed consent.

### RNA-seq

For circRNAs sequencing, total RNA was extracted from six pairs of frozen melanoma tissues using TRIzol Reagent (Life, CA, USA). Subsequently, the RNA underwent treatment with a Ribo-off rRNA Depletion Kit (Vazyme, China) to eliminate ribosomal RNA prior to the generation of the RNA-seq library. Following this, an Illumina Novaseq^TM^6000 instrument (Illumina, USA) was utilized for library sequencing. The FASTQ reads were aligned to the human reference genome (hg38/GRCh38). Subsequently, the counts of the remaining reads were normalized and mapped across an identified back-splice junction.

For mRNA sequencing, mRNA with poly(A) was purifed, fragmented, and then reverse-transcribed to generate the RNA-seq library. Finally, sequencing was conducted using an Illumina Novaseq^TM^6000 system (Illumina, USA). The resulting reads were mapped to the genome.

### Cell culture and transfection

The melanoma cell lines A375, A2058, MV3, Sk-mel-1, Sk-mel-28, and the normal epidermal melanocyte cell line PIG1 were procured from the Cell Bank of the Chinese Academy of Sciences (Shanghai, China). These cells were cultured at 37 °C with 5% CO_2_ and were confirmed to be free of mycoplasma contamination. The human circFCHO2 cDNA was synthesized and inserted into the pcDNA3.1( +) Laccase2 MCS Exon Vector by Hanbio Co., Ltd. (Shanghai, China). An empty vector was employed as the negative control. Two small interfering RNA (siRNA) sequences were synthesized, and a scramble siRNA was synthesized as a negative control. The transfected cells underwent screening with puromycin (2 μg/ml) for 1 week to establish stable cell lines. Transient transfection was carried out using Lipofectamine 2000 (Invitrogen, Carlsbad, CA, USA) following the manufacturer’s instructions. Total proteins and RNA were collected 48 h post-transfection. Plasmids and siRNAs of DND1 were synthesized by Genomeditech Co., Ltd. (Shanghai, China) and tranfected as described above. The specific target sequences utilized in this study are detailed in Table S1.

### Quantitative real‑time PCR and western blot assays

QRT-PCR and western blot analyses were conducted following the procedures outlined in our previous study. Detailed protocols can be found in the Supplementary Materials and Methods (Wei et al. 2019a). The primers and antibodies used in the study are provided in Table S2 and S3, respectively.

### *Immunohistochemistry and fluorescence *in situ* hybridization assays*

Immunohistochemistry (IHC) assay was conducted following the procedures outlined in our previous study. The detailed protocol can be found in the Supplementary Materials and Methods (Wei et al. 2019b). The antibodies employed in this study are listed in Table S3. For the fluorescence in situ hybridization (FISH) assay, probes with 3′-Cy3 modifcation targeting circFCHO2 and ICC-labeled probes specific to DND1 were synthesized by Geneseed Biotech Co., Ltd. (Guangzhou, China), respectively. The FISH probe sequence employed in this study is provided in Table S4. Cells were initially fixed with 4% paraformaldehyde and subsequently incubated with probes over night at 37℃. Nuclei were counterstained with DAPI and captured using confocal microscopy (Carl Zeiss, Germany).

### RNA-pull down assay

A biotin-labeled circFCHO2 probe (RiboBio, China) was incubated with streptavidin magnetic beads (RioBio, China) at room temperature for 2 h to generate probe-coated beads. Lysates from A375 and MV3 cells were incubated with probe-coated beads at 4℃ overnight. Subsequently, the beads were washed, and the proteins pulled down were analyzed using silver staining, mass spectrometry, and western blotting. The RNA Pulldown probe sequence utilized in this study is provided in Table S5.

### RNA immunoprecipitation assay

RNA immunoprecipitation (RIP) assay was conducted using a Magna RIP RNABinding Protein Immunoprecipitation Kit (Millipore, Billerica, MA, USA), following the procedures outlined in our previous study (Zhang et al. 2019). Biotin-labeled circFCHO2 was synthesized by Geneseed Biotech (Guangzhou, China). In brief, circFCHO2-overexpressing cells were washed with PBS, fixed in 1% formaldehyde, lysed in Co-IP buffer, subjected to sonication, and finally centrifuged. A portion of the supernatant (50μL) was reserved as input, while the remaining supernatant was incubated with a mixture of probes-streptavidin-dynabeads (M-280; Invitrogen, Carlsbad, CA, USA) at 30℃ for 12 h. The mixture underwent further incubation with lysis buffer and proteinase K. Subsequently, RNA was extracted using TRIzol Reagent (Invitrogen, Carlsbad, CA, USA), eluted, reverse transcribed to cDNA, and subsequently detected by qRT-PCR.

### RNase R treatment and actinomycin D assays

RNase R (Lucigen, USA) was employed for RNA digestion. Briefy, RNAs extracted from A375 and MV3 cells were divided into two fractions. For RNase R digestion, 1 µg of RNA was treated with 2U of RNase R. As a control, 1 µg of RNA was mixed with an equal volume of RNase-free water. Subsequently, the expression levels of circFCHO2 and FCHO2 mRNA were assessed through qRT-PCR. CircFCHO2 stability assays involved seeding melanoma cells into 12-well plates, followed by treatment with medium containing actinomycin D (5 μg/mL) (Genview, Beijing, China) or DMSO for 0, 3, 6, and 9 h.

### Nuclear‑cytoplasmic fractionation assays

RNA from nuclear and cytoplasmic fractions of melanoma cells was separated using a commercial Paris kit (Life Technologies, Gaithersburg, MD, USA) following the manufacturer’s instructions. CircFCHO2, FCHO2, and GAPDH cDNA were synthesized using a PrimeScript RT Master Mix (Takara) while snoU6 cDNA was generated through stem-loop methods (RiboBio, Guangzhou, China). The expression levels of circFCHO2, FCHO2, GAPDH, and snoU6 in the nuclear and cytoplasmic fractions were determined by qRT-PCR.

### Colony formation, CCK-8, transwell invasion, and wound healing migration assays

Colony formation, CCK-8, transwell invasion, and wound healing migration assays were conducted following the methodologies outlined in our previous studies. Detailed protocols can be found in the Supplementary Materials and Methods (Wei et al. 2018; Zhu et al. 2019).

### FCM assays of the cell cycle and apoptosis

The transfected cells underwent staining with propidium iodide (KGA512, KeyGen Biotech, Nanjing, China), and the cell cycle was evaluated using CytoFLEX FCM (Beckman Coulter, Brea, CA, USA). The percentages of cells in G0/G1, S, and G2 phases were determined and compared. For apoptosis detection, cells were stained with an Annexin V-FITC Apoptosis Detection Kit (Keygen Biotech), and apoptosis was assessed by CytoFLEX FCM. The ratio of early to late apoptotic cells was calculated.

### In vivo* assays*

All animal experiments received approval from the Animal Experimentation Ethics Committee of Zhongshan Hospital, Fudan University. Four-week-old male nude mice (BALB/c) were housed and cared for in accordance with the principles of the 3Rs (replacement, reduction, and refinement). A total of 5 × 10^6^ cells, resuspended in 100μL of PBS, were subcutaneously inoculated into the left flank of each mouse. Following tumor detection, measurements of tumor size were taken every 3 days using a vernier caliper, and tumor volume was calculated using the formula volume (cm^3^) = *L* × *W*^2^ × 0.5, where *L* and *W* denote the largest and smallest diameters, respectively. Euthanasia was performed when the tumor volumes reached a maximum of 2000mm^3^.

### Statistical analysis and the resources of public data

Gene expression and survival data of melanoma cohorts were obtained from The Cancer Genome Atlas (TCGA)-SKCM (data format: TPM. Workflow: STAR) DataSet (https://www.cancer.gov/ccg/research/genome-sequencing/tcga). The secondary structure of circFCHO2 was obtained in the CSCD (Cancer Specific CircRNA Database, http://gb.whu.edu.cn/CSCD/) database. The experimental data were processed and subjected to statistical analysis using SPSS software (version 23.0) and GraphPad Prism software (version 8.0). Continuous measurement data are expressed as mean ± standard deviation (mean ± SD), while categorical measurement data are presented as quantity and percentage. Two sets of continuous econometric data were subjected to Student’s *t*-test or paired *t*-test. The Mann–Whitney *U* test was employed for the analysis of several types of measurement data. Survival analysis was conducted using the Kaplan–Meier method, generating survival curves for each group, with differences in survival between groups assessed by the log-rank test. Furthermore, Cox proportional risk regression models were utilized for univariate and multivariate correlation analysis of independent prognostic factors. In this study, three independent replicates were performed for different experiments and the experimental measurement data were uniformly standardized. In all statistical analyses presented in this experiment, a difference was deemed statistically significant when *P < 0.05, **P < 0.01, or ***P < 0.001.

## Results

### Identification and characterization of circFCHO2 in melanoma tissues and cells

**Fig. 1:**
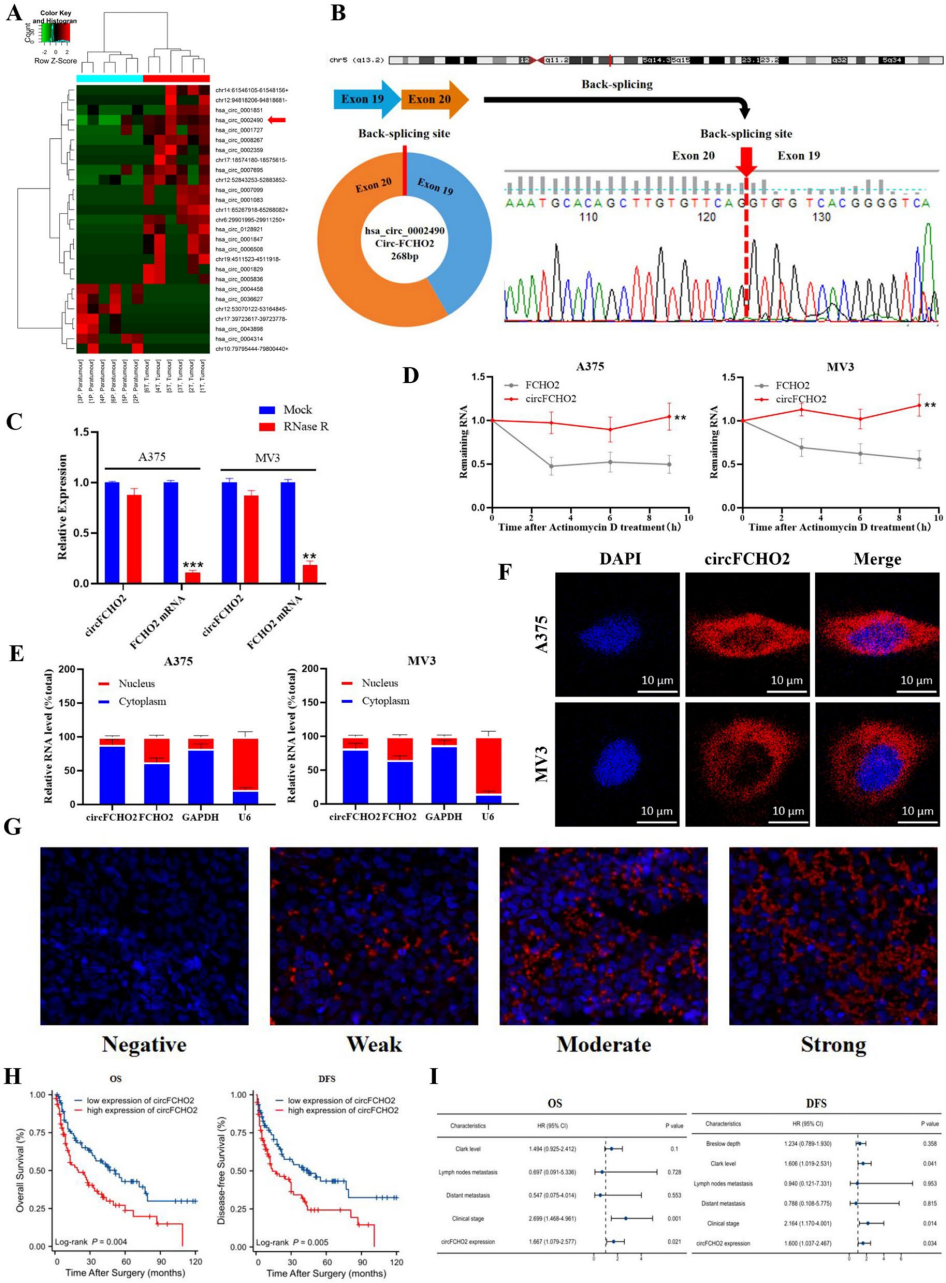
Identification and characterization of circFCHO2 in melanoma cells and tissues. **A** Clustered heatmap of the differentially expressed circRNAs in six melanoma and paracancerous tissues. **B** Schematic illustration of the genomic location and back-splicing of circFCHO2, with the splicing site validated by Sanger sequencing. **C** CircFCHO2 and FCHO2 mRNA expression in melanoma cells were detected after RNase R treatment compared to mock treatment. **D** Stabilities of circFCHO2 and FCHO2 in A375 and MV3 cells treated with actinomycin D. **E** qRT-PCR for the distribution of FCHO2, circFCHO2, U6, and GAPDH in the cytoplasmic and nuclear fractions of A375 and MV3 cells. **F** The subcellular location of circFCHO2 in melanoma cells was investigated by FISH. **G** Representative micrographs of circFCHO2 expression within melanoma. **H**–**I** Kaplan–Meier survival curves and forest plots for OS and DFS of patients with melanoma. Values are expressed as the means ± SDs; *P < 0.05; **P < 0.01; ***P < 0.001; ns, no significant

To find the most important differentially expressed circular RNA in melanoma, we screened 26 significantly differentially expressed circular RNAs (Fig. 1A) by next-generation RNA sequencing of 6 pairs of tumor tissues and paired paracancerous tissues from melanoma surgical specimens collected previously. After comparison with the circBase database (http://www.circbase.org/), we identified 16 circular RNAs whose numbers and sequences can be queried in the database, and further used qRT-PCR to detect the expression level of these significantly different circular RNAs in melanoma tumor tissues (*n* = 9) and paracancerous tissues (*n* = 9). The results show that hsa_circ_0002490 (named circFCHO2) showed the most significant difference in expression compared to the other circular RNAs detected (Fig. S1A). qRT-PCR was employed to detect the expression level of circFCHO2 in both melanoma and benign nevus tissue samples (Fig. S1B).The results indicated a significantly higher expression of circFCHO2 in melanoma tissue compared to benign nevus tissue. Based on the detection results in clinical tissue samples, we further verified whether the expression of circFCHO2 at the cytological level was consistent with the histological trend. The qRT-PCR analysis revealed that the expression of circFCHO2 was higher in the five melanoma cell lines compared to the human epidermal melanoma cell line (Fig. S1C).

We performed Sanger sequencing on circFCHO2 to investigate its cyclization properties. The sequencing results showed that circFCHO2 is derived from exons 19 and 20 of the FCHO2 gene on chromosome 5, with a length of 268 bp. It is a circular RNA formed by back-splicing the pre-mRNA of FCHO2, which links the first and last exons 19 and 20 of FCHO2 (Fig. 1B). Moreover, circFCHO2 expression remained significantly unchanged in A375 and MV3 cells after treatment with RNase R, suggesting an intact circular structure. In contrast, the expression of FCHO2 mRNA was significantly downregulated in the melanoma cell lines (Fig. 1C). In order to further validate the stability of the circFCHO2 structure, actinomycin D assay was conducted. The results demonstrated that the T_1/2_ of circFCHO2 was greater than that of FCHO2 mRNA in A375 and MV3 cells treated with actinomycin D (Fig. 1D). Circular RNA’s biological functions and specific mechanism of action are intricately tied to its cellular location. In our study, we observed that circFCHO2 was predominantly localized in the cytoplasm of melanoma cells (Fig. 1E, F).

To explore the clinical significance of circFCHO2, we further detected the expression of circFCHO2 in tissue microarray (tumor tissue from 158 patients with melanoma) using fluorescence probe of in situ hybridization, and divided it into low and high expression groups based on fluorescence intensity (Fig. 1G) (low expression group *n* = 78 cases, high expression group *n* = 80 cases), and combined with clinical information indicators to perform prognostic analysis. Statistical results revealed significant correlations between circFCHO2 expression and various clinical parameters, including anatomical site (*P* = 0.003), histological classification (*P* = 0.008), lymph node metastasis (*P* = 0.002), distal metastasis (*P* = 0.012), and clinical staging (*P* < 0.001) (Table 1). The results of Kaplan–Meier survival analysis demonstrated that melanoma patients with high circFCHO2 expression (OS&DFS) had a poorer prognosis compared to patients with low circFCHO2 expression (Fig. 1H). The results of univariate analysis demonstrated that Clark grade, lymph node metastasis, distal metastasis, clinical stage, and circFCHO2 expression level were negatively correlated with overall survival of melanoma patients after surgery, while Breslow thickness, Clark grade, lymph node metastasis, distal metastasis, clinical stage, and circFCHO2 expression level were negatively correlated with progression-free survival of melanoma patients after surgery. Multivariate analysis results suggested that several indicators, including circFCHO2 expression level, could serve as independent risk factors for predicting the prognosis of melanoma patients (Table 2 and Fig. 1I). Taken together, these findings suggest that circFCHO2 is a circular RNA, which is upregulated in melanoma tissues and exhibiting predominant localization in the cytoplasm of melanoma cells.

**Table 1:** Correlations between circFCHO2 expression and clinicopathologic features in 158 melanoma patients

Variable	Patients		circFCHO2 expression		
	No	%	Low	High	*P*-value*
All patients	158	100.0	78	80	
Age					0.112
< 60	65	41.1	37	28	
≥ 60	93	58.9	41	52	
Gender					0.154
Male	82	51.9	36	46	
Female	76	48.1	42	34	
Atomic site					0.003
Acra	82	51.9	51	31	
Trunk	40	25.3	13	27	
Other	36	22.8	14	22	
Histologic type					0.008
Superficial spreading	40	25.3	13	27	
Nodular	32	20.3	15	17	
Acral	50	31.6	34	16	
Lentigo maligna	36	22.8	16	20	
Breslow depth (mm)					0.203
≤ 2	79	50	43	36	
> 2	79	50	35	44	
Clark level					0.039
I–III	78	49.4	45	33	
IV–V	80	50.6	33	47	
Ulceration					0.057
Present	22	13.9	15	7	
Absent	136	86.1	63	73	
Lymph node metastasis					0.002
No	134	84.8	73	61	
Yes	24	15.2	5	19	
Distant metastasis					0.012
No	127	80.4	69	58	
Yes	31	19.6	9	22	
Clinical stage					< 0.001
I–II	105	66.5	64	41	
III–IV	53	33.5	14	39	

^*^P < 0.05 was regarded as statistically significant, *P* value was calculated using Cox proportional hazards regression

**Table 2:** Univariate and multivariate analyses of prognostic factors associated with OS and DFS

Variable	Overall survival			Disease-free survival		
	Univariate *P*-value	Multivariate *P*-value	Multivariate HR (95% CI)	Univariate *P*-value	Multivariate *P*-value	Multivariate HR (95% CI)
Age, year (≥ 60 vs. < 60)	0.515	NA	NA	0.825	NA	NA
Gender (male vs. female)	0.405	NA	NA	0.494	NA	NA
Anatomic site (acra vs. trunk vs. other)	0.739	NA	NA	0.949	NA	NA
Histologic type (superficial reading vs. nodular vs. acral vs. lentigo maligna)	0.056	NA	NA	0.104	NA	NA
Breslow depth (mm) (> 2 vs. ≤ 2)	0.088	NA	NA	0.032	0.358	1.234 (0.789–1.930)
Clark level (IV–V vs. I–III)	0.001	0.100	1.494 (0.925–2.412)	0.001	0.041	1.606 (1.019–2.531)
Ulceration (present vs. absent)	0.160	NA	NA	0.176	NA	NA
Lymph node metastasis (yes vs. no)	< 0.001	0.728	0.697 (0.091–5.336)	< 0.001	0.953	0.940 (0.121–7.331)
Distant metastasis (yes vs. no)	0.003	0.553	0.547 (0.075–4.014)	0.007	0.815	0.788 (0.108–5.775)
Clinical stage (III–IV vs. I–II)	< 0.001	0.001	2.699 (1.468–4.961)	< 0.001	0.014	2.164 (1.170–4.001)
circFCHO2 expression (high vs. low)	0.005	0.021	1.667 (1.079–2.577)	0.006	0.034	1.600 (1.037–2.467)

A chi-square test was used for comparing groups between low and high circFCHO2 expression. *P < 0.05 was considered significant

### *circFCHO2 promotes the proliferation, migration, and invasion of melanoma cells *in vitro

**Fig. 2:**
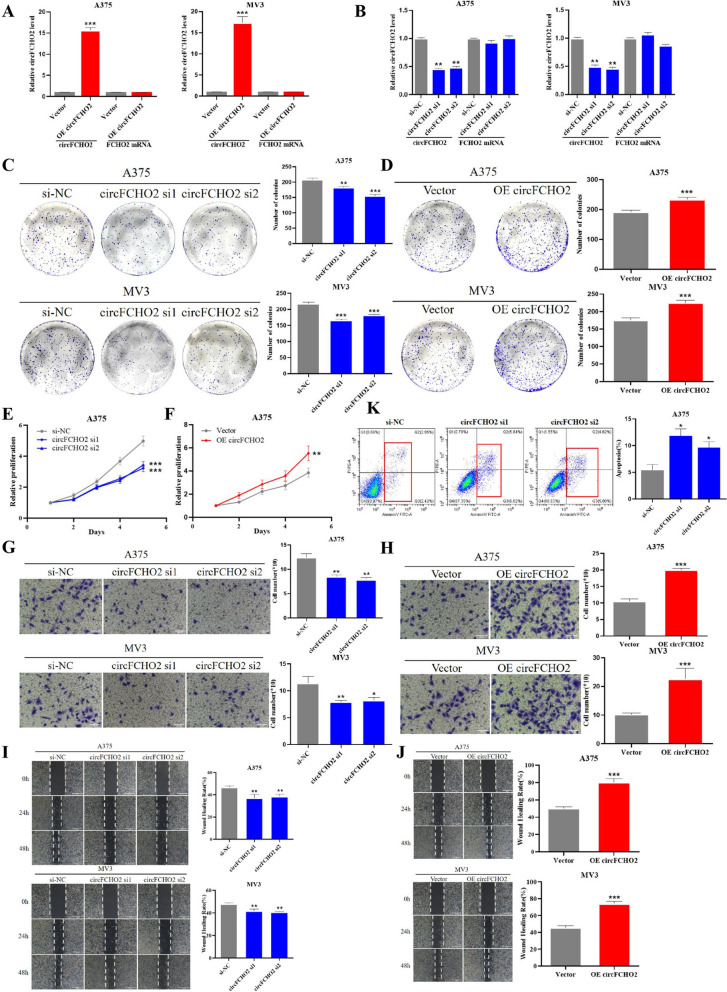
circFCHO2 promotes the proliferation, migration, and invasion of melanoma cells in vitro. **A**, **B** qRT-PCR analysis of circFCHO2 and FCHO2 mRNA expression in A375 and MV3 cells transfected with circFCHO2 siRNAs or circFCHO2 overexpression vectors. **C**, **D** Colony formation assay was used to detect the proliferation ability of melanoma cells with different treatments. **E**, **F** CCK-8 assay was performed to detect the proliferation of melanoma cells. **G**, **H** Transwell invasion assay was used to detect the invasion ability of melanoma cells following different treatments. **I**, **J** Wound healing migration assay was performed to detect the migration ability of melanoma cells with different treatments. The migration of A375 and MV3 cells was photographed at 0 h, 24 h, and 48 h. **K** Effect of circFCHO2 on apoptosis as determined by Annexin V-FITC staining and FCM detection. Unpaired Student’s *t*-test, Mann–Whitney *U* test, Kruskal–Wallis test and one-way ANOVA test were used for the statistical analyses. *P < 0.05; **P < 0.01; ***P < 0.001

To investigate the effect of circFCHO2 on the biological function of melanoma, we transiently transfected human melanoma A375 and MV3 cell lines with circFCHO2 interference fragment (siRNA), and transfected and constructed A375 and MV3 cell lines stably overexpressing circFCHO2 with lentivirus vector without affecting the expression of FCHO2 mRNA. qRT-PCR was utilized to detect the expression level of circFCHO2 to verify the efficiency of knockdown and overexpression of circFCHO2. The results demonstrated that the overexpression efficiency of stable transgenic strains overexpressing circFCHO2 was 15–20 times higher than that of the control group (Fig. 2A), and the interference efficiency of interference fragments of circFCHO2 was more than 50% compared to the control group (Fig. 2B).

Clone formation and CCK-8 assays demonstrated that the number of clones formed in A375 and MV3 cells significantly decreased by circFCHO2 knockdown and increased by circFCHO2 overexpression compared to the control group (Fig. 2C–D). CCK-8 assays showed that circFCHO2 knockdown in A375 cells significantly reduced the absorbance at 450 nm (Fig. 2E) while circFCHO2 overexpression significantly increased the absorbance at 450 nm compared to the control group (Fig. 2F). The transwell invasion assays demonstrated that circFCHO2 knockdown significantly reduced the number of A375 and MV3 cells penetrating the transwell compartment (Fig. 2G) while circFCHO2 overexpression increased the number of A375 and MV3 cells penetrating the transwell compartment compared to the control group (Fig. 2H). The results of the wound healing migration assay demonstrated that the proportion of A375 and MV3 cell scratch areas recovered significantly decreased by circFCHO2 knockdown (Fig. 2I) and increased by circFCHO2 overexpression compared to the control group (Fig. 2J). To further clarify the factors associated with the pro-cancer phenotype, we further detected melanoma cells overexpressing circFCHO2 by flow cytometry. The experimental results showed that the apoptosis rate of A375 cell knockdown circFCHO2 was significantly higher than that of the control group (Fig. 2K), while the overexpression of circFCHO2 did not lead to a significant alteration in the distribution of cells across each phase of the cell cycle in A375 cells when compared to the control group (Fig. S2A). Taken together, these findings illustrated the oncogenic role of circFCHO2 in melanoma cells.

### *circFCHO2 facilitates the tumorigenesis of melanoma cells *in vivo* and the PI3K/AKT signaling pathway*

**Fig. 3:**
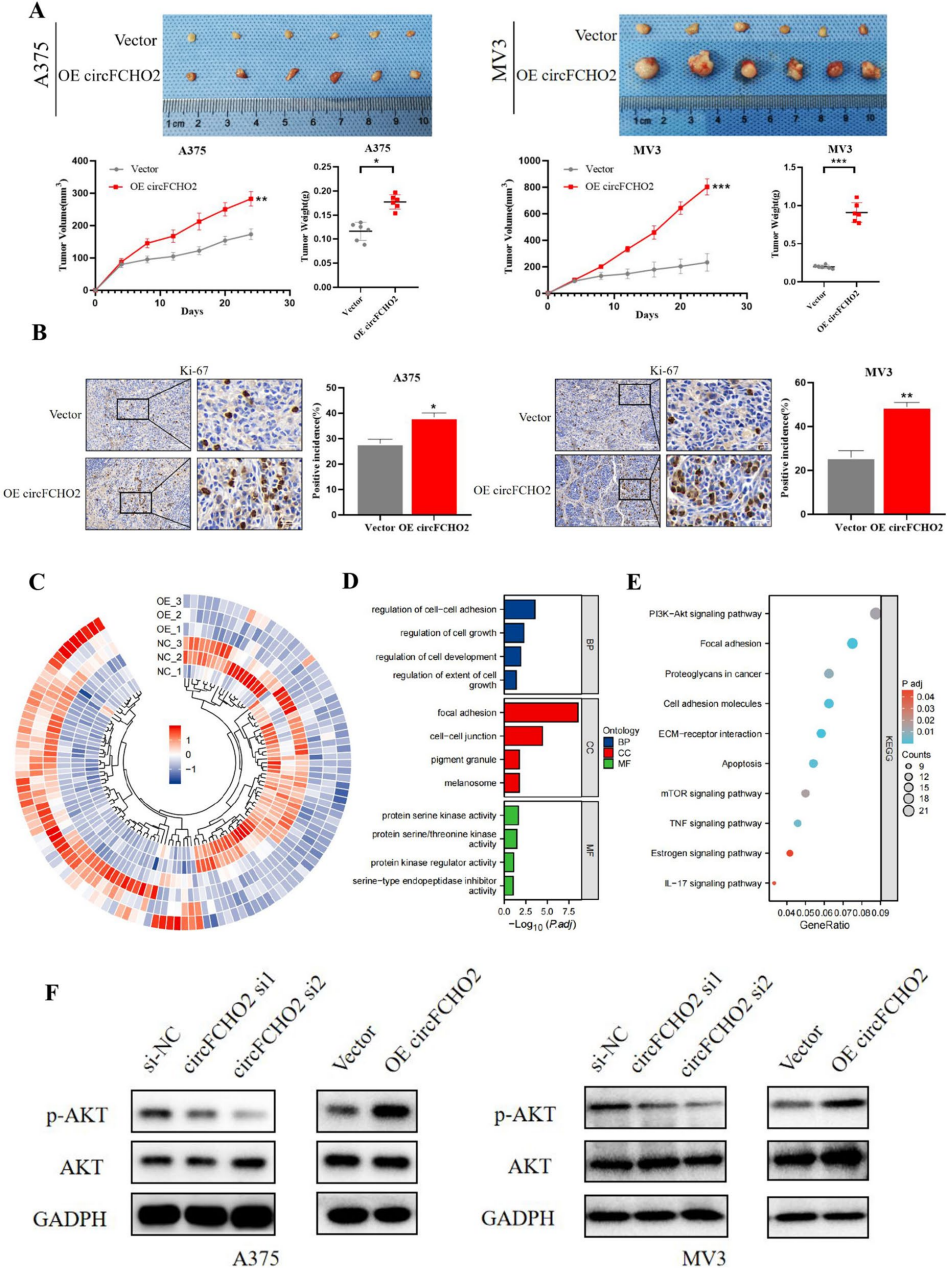
circFCHO2 facilitates the tumorigenesis of melanoma cells in vivo and the PI3K/AKT signaling pathway. **A** Representative picture of subcutaneous xenograft tumors (*n* = 6 for each group). Curves of tumor volumes and weights show positive effects of circFCHO2 overexpression on the formation of subcutaneous xenograft tumors. **B** Ki-67 IHC staining of xenograft tumors and H-scores of the Ki67 staining. **C** The circos heatmap of differentially expressed mRNAs in A375 cells transfected with Vector or circFCHO2. Each sample was mixed with three replicates. **D**, **E** GO and KEGG analyses of differentially expressed mRNAs in A375 cells. **F** Western blot assay was used to detect the p-AKT and AKT levels in melanoma cells with different treatment, GAPDH was used as a negative control. Paired Student’s *t*-test, unpaired Student’s *t*-test, Mann–Whitney *U* test, one-way ANOVA test, and Kruskal–Wallis test were used for the statistical analyses.*P < 0.05; **P < 0.01; ***P < 0.001

To further verify whether circFCHO2 can affect the growth ability of melanoma cells in vivo, we performed subcutaneous melanoma inoculation in nude mice and compared the final subcutaneous tumor situation. The tumors were then excised for immunohistochemical staining of the Ki-67 protein. The experimental results showed that the volume and weight of A375 and MV3 cells in the subcutaneous tumourigenesis of nude mice were significantly higher by circFCHO2 overexpression than those of the control group (Fig. 3A), and the positive rate of Ki-67, a cell proliferation marker, in the subcutaneous melanoma of nude mice in the circFCHO2 overexpression group was significantly higher than that of the control group (Fig. 3B).

In order to delve deeper into its precise mechanism of action, we conducted transcriptome sequencing on the human melanoma A375 cell line stably overexpressing circFCHO2 and the empty control A375 cell line. The analysis results demonstrated that the PI3K/AKT pathway was the most significant among the pathways enriched by differential genes (Fig. 3C–E). Therefore, we speculate that the major molecular signaling pathway through which circFCHO2 induces changes in biological function in melanoma cells is the PI3K/AKT signaling pathway. We used Western blot assay to verify the results, which showed that the expression of phosphorylated AKT (pAKT) increased in A375 cells and MV3 cell lines overexpressing circFCHO2 compared to the control group, but the total expression of AKT did not change significantly. The expression of pAKT in the circFCHO2 knockdown group decreased compared to the control group, while the total AKT expression did not show significant changes (Fig. 3F, G). These results suggest that circFCHO2 can promote melanoma proliferation in vivo and affect the PI3K/AKT signaling pathway.

### circFCHO2 physically interacts with DND1

**Fig. 4:**
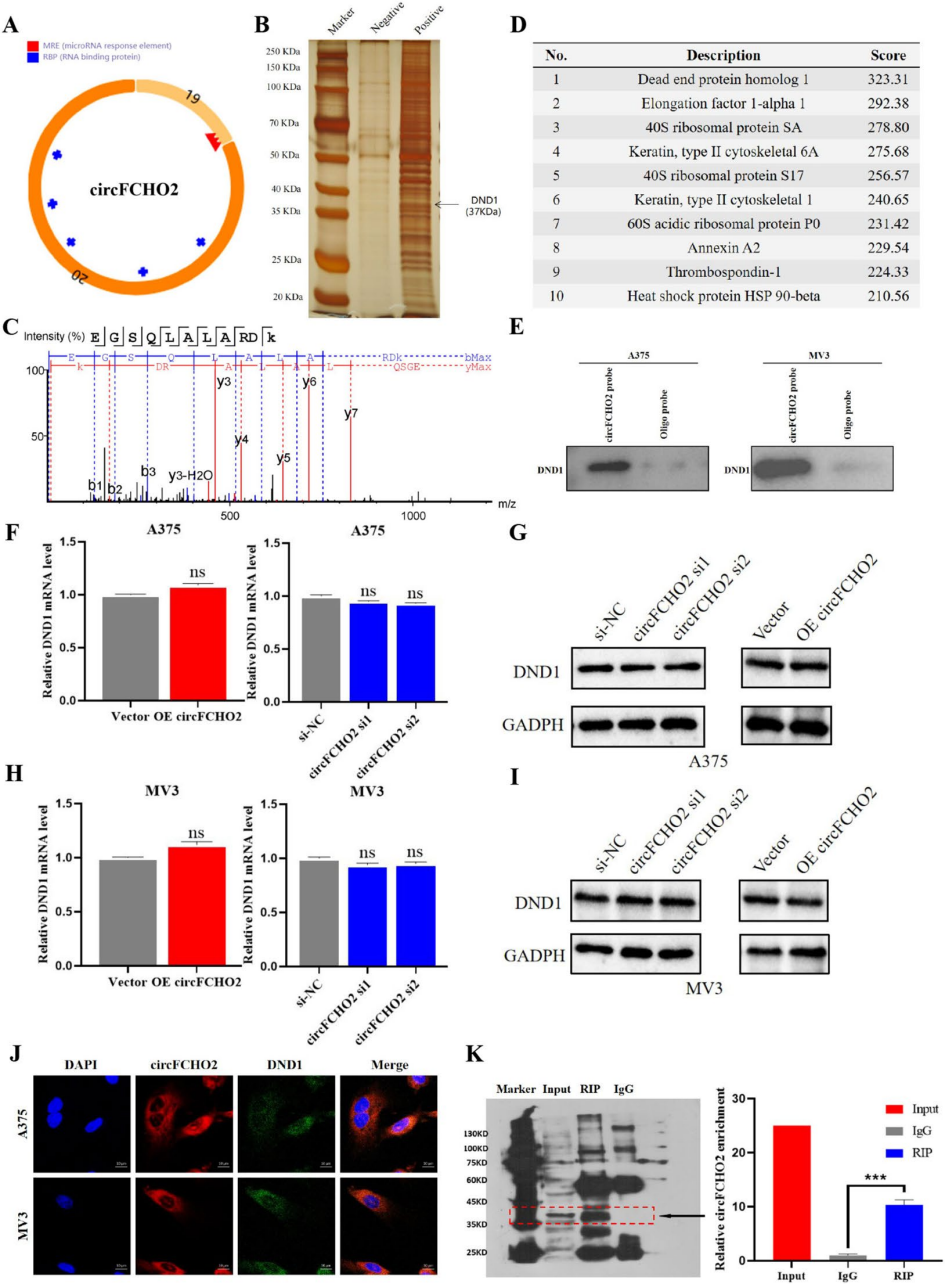
circFCHO2 physically interacts with DND1. **A** The visualization structure of circFCHO2 in the CSCD database (http://gb.whu.edu.cn/CSCD/). The blue mark represents the RBP binding site while the red mark represents the miRNA binding site. **B** Silver staining image of RNA pull-down assay with circFCHO2 (positive) and control (negative) probes. **C**, **D** Mass spectrometry analysis of circFCHO2-binding proteins after RNA pull-down assay and list of top ten differentially expressed proteins identified by mass spectrometry. **E** Levels of DND1 protein were detected in the proteins pulled down by circFCHO2 and oligo probes. **F**–**I** Levels of DND1 mRNA and protein in circFCHO2-overexpressing and circFCHO2-knockdown melanoma cells were quantified by qRT-PCR and western blotting. GADPH served as the internal control. **J** Representative images of circFCHO2 and DND1 colocalization in melanoma cells detected by FISH assay. **K** RIP assays revealing circFCHO2 enrichment by DND1 in A375 cells. Unpaired Student’s *t*-test and Mann–Whitney *U* test were used for the statistical analyses. ***P < 0.001; ns, no significant

We searched the secondary structure of circFCHO2 in the CSCD (Cancer Specific CircRNA Database, http://gb.whu.edu.cn/CSCD/) database and found that, unlike most other circular RNA structures, it has fewer miRNA binding sites and more RNA binding protein binding sites (Fig. 4A). Therefore, we speculate that circFCHO2 may have a binding function with RBP. To verify our conjecture, we designed a circFCHO2 probe to pull down all protein molecules that can bind to circFCHO2 by RNA pulldown experiment. Compared with the control group, the biotinylated circFCHO2 group has a distinct band with a molecular weight of 35–40 kDa (Fig. 4B), which was identified as DND1 by mass spectrometry (Fig. 4C–E). Therefore, it is tentatively suggested that DND1 is the RBP most likely to bind to circFCHO2. The results of qRT-PCR and Western blot revealed that circFCHO2 did not exert a significant impact on the mRNA and protein levels of DND1 (Fig. 4F–I). We then prepared circFCHO2 fluorescence in situ hybridization probe and performed fluorescence in situ hybridization co-localization (FISH) experiment to observe the co-localization of DND1 and circFCHO2 in cells. The results showed that circFCHO2 and DND1 had obvious co-localization in A375 and MV3 cells (Fig. 4J). To further confirm whether circFCHO2 is bound to DND1, we used RNA binding protein immunoprecipitation experiment to verify. Western blot results showed that the IgG group could not bind DND1, and the immunoprecipitation successfully bound DND1, and compared with the IgG group, the amount of DND1 bound by the RIP group was more significant (Fig. 4K). The detection results of RNA products precipitated from the RIP experiment by qRT-PCR showed that the circFCHO2 content in the RIP group was more than 10 times higher than that in the IgG group, with significant statistical differences. Overall, these findings indicate a physical interaction between circFCHO2 and DND1 in melanoma cells.

### DND1 inhibits progression of melanoma by mediating the PI3K/AKT signaling pathway

**Fig. 5:**
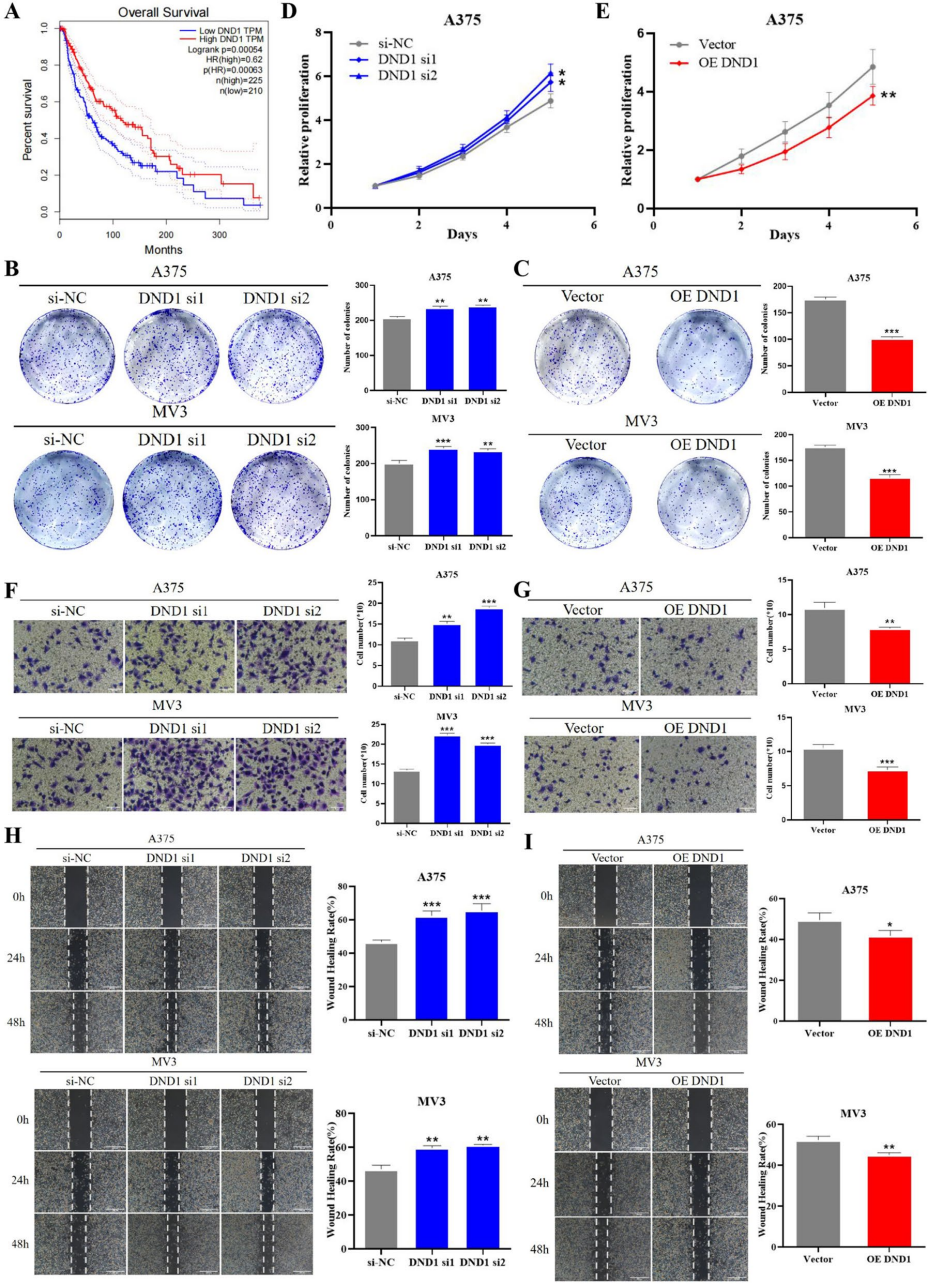
DND1 inhibits progression of melanoma by mediating the PI3K/AKT signaling pathway. **A** Based on the expression of DND1, the overall survival curve was performed using Kaplan–Meier methods and analyzed by the log-rank test. Gene expression and survival data of melanoma cohorts were obtained from The Cancer Genome Atlas (TCGA)-SKCM(data format: TPM. Workflow: STAR) DataSet (https://www.cancer.gov/ccg/research/genome-sequencing/tcga). **B**, **C** Colony formation assay was used to detect the proliferation ability of melanoma cells with different treatments. **D**, **E** CCK-8 assay was performed to detect the proliferation of melanoma cells. **F**, **G** Transwell invasion assay was used to detect the invasion ability of melanoma cells following different treatments. **H**, **I** Wound healing migration assay was performed to detect the migration ability of melanoma cells with different treatments. The migration of A375 and MV3 cells were photographed at 0 h, 24 h, and 48 h. Unpaired Student’s *t*-test, Mann–Whitney *U* test, Kruskal–Wallis test, and one-way ANOVA test were used for the statistical analyses. *P < 0.05; **P < 0.01; ***P < 0.001

To investigate whether DND1 and circFCHO2 affect the biological function of melanoma, we further investigated the biological function of DND1 in melanoma. Initially, survival analysis was conducted using the SKCM (cutaneous melanoma) melanoma dataset (data format: TPM. Workflow: STAR) in the TCGA database (https://www.cancer.gov/ccg/research/genome-sequencing/tcga). The outcomes revealed a significantly shorter overall survival in the DND1 low-expression group (*n* = 210) compared to the DND1 high-expression group (*n* = 225). The Logrank test yielded a *P*-value of 0.00054, which is less than the threshold of 0.001, indicating a statistically significant difference. The hazard ratio (HR) value is 0.62, indicating a 38% reduction in the risk of death in the high DND1 expression group compared to the low DND1 expression group, *P* = 0.00063 < 0.001, and the difference is statistically significant (Fig. 5A). It is speculated that DND1 might function as an inhibitory molecule in the progression of melanoma.

To verify our conjecture based on the results of bioinformatics analysis, we proceeded to examine the regulatory impact of DND1 on the in vitro proliferation of melanoma cells. qRT-PCR and Western blot assays confirmed successful overexpression and knockdown of DND1 in A375 and MV3 cell lines (Fig. S3A-D). Clone formation experiment demonstrated that the number of clones formed in A375 and MV3 cells was significantly increased by DND1 knockdown (Fig. 5B) and decreased by DND1 overexpression compared to the control group (Fig. 5C). CCK-8 assays showed that the absorbance value at 450 nm was significantly increased by DND1 knockdown (Fig. 5D) and decreased by DND1 overexpression in A375 cells compared to the control group (Fig. 5E). Transwell invasion assays revealed a substantial increase in the number of A375 and MV3 cells penetrating the transwell compartment upon DND1 knockdown (Fig. 5F), while DND1 overexpression resulted in a reduction compared to the control group (Fig. 5G). Scratch assays showed that the proportion of recovered scratch areas of A375 and MV3 cells was significantly increased by DND1 knockdown (Fig. 5H) and reduced by DND1 overexpression compared to the control group (Fig. 5I).

According to the previous research reports, DND1 can regulate PI3K/AKT, TGF, WNT, and other signaling pathways and play a role in relevant evidence (Yamaji et al. 2017); we speculate that the effect of DND1 on the biological function of melanoma may also be achieved by regulating the PI3K/AKT signaling pathway. Therefore, we applied Western blot assay to verify, and the results demonstrated that the expression of phosphorylated AKT (pAKT) in A375 cells and MV3 cell lines overexpressing DND1 decreased compared with the control group, but the total AKT expression did not exhibit a significant change, indicating that overexpression of DND1 downregulated the PI3K/AKT signaling pathway in melanoma cells. Compared with the control group, the expression of pAKT increased in the DND1 knockdown group, while the total expression of AKT did not change significantly (Fig. S3E), indicating that DND1 knockdown upregulated the PI3K/AKT signaling pathway in melanoma cells. So far, we can tentatively confirm that DND1 inhibits the PI3K/AKT signaling pathway in melanoma.

### Elevated DND1 turnovers the circFCHO2-induced malignant phenotype of melanoma

**Fig. 6:**
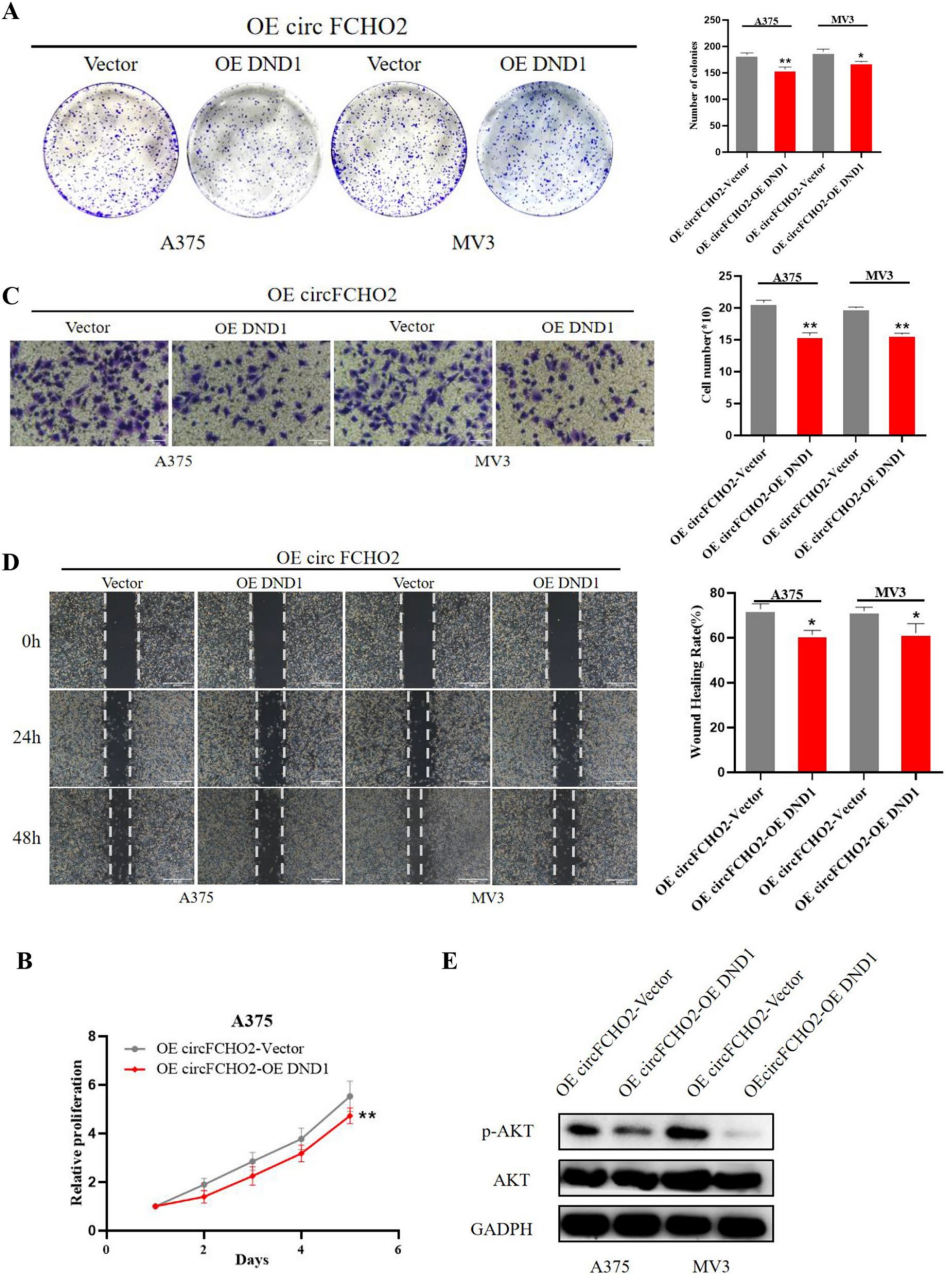
Elevated DND1 turnovers the circFCHO2-induced malignant phenotype of melanoma. **A** Colony formation assay was used to detect the proliferation ability of melanoma cells with different treatments. **B** CCK-8 assay was performed to detect the proliferation of melanoma cells. **C** Transwell invasion assay was used to detect the invasion ability of melanoma cells following different treatments. **D** Wound healing migration assay was performed to detect the migration ability of melanoma cells with different treatments. The migration of A375 and MV3 cells were photographed at 0 h, 24 h, and 48 h. E. Western blot assay was used to detect the p-AKT and AKT levels in melanoma cells with different treatment, GAPDH was used as a negative control. Paired Student’s *t*-test, unpaired Student’s *t*-test, Mann–Whitney *U* test, one-way ANOVA test, and Kruskal–Wallis test were used for the statistical analyses.*P < 0.05; **P < 0.01

**Fig. 7:**
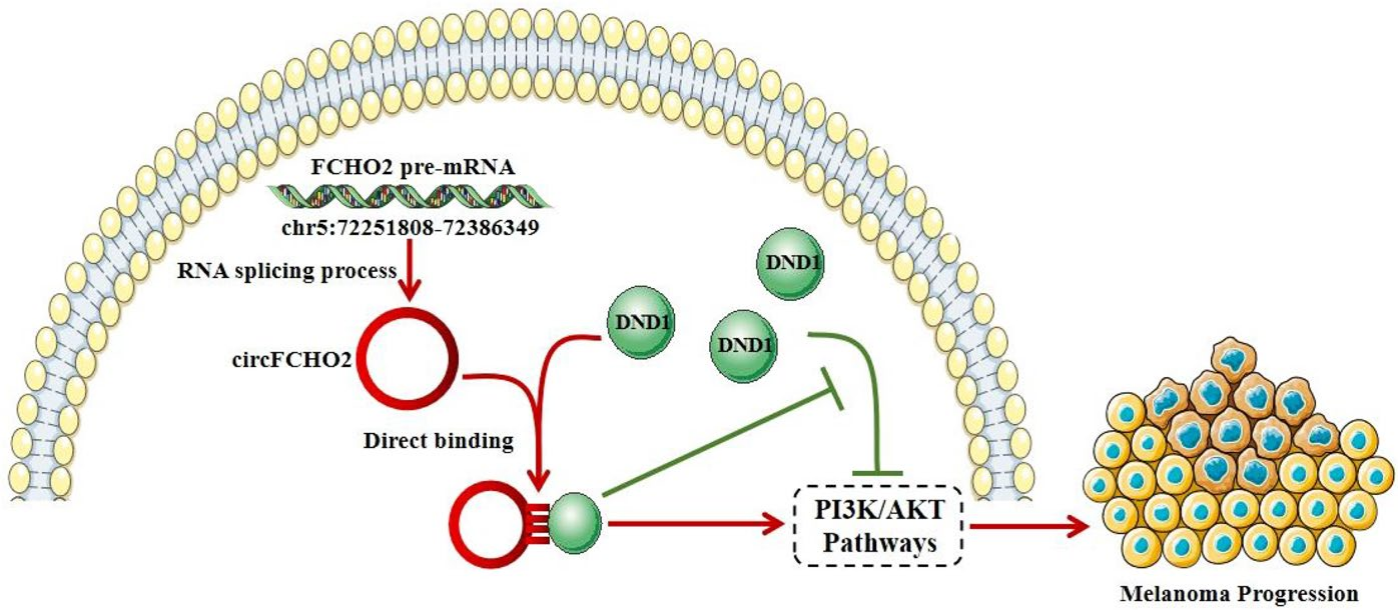
Schematic summary of the mechanism by which circFCHO2 promotes the proliferation of melanoma cells

Previous studies have reported that by binding to a protein, circular RNA can block its function of interacting with another protein (Fang et al. 2018; Xueting et al. 2019; Bi et al. 2018; Zhu et al. 2020; Shen et al. 2020; Zhao et al. 2020; Lou et al. 2020). Based on the previous experimental results, we speculate that circFCHO2 also blocks the interaction between DND1 and signaling pathway proteins by this mechanism, thereby inhibiting its downregulating effect on the PI3K/AKT signaling pathway. To confirm that circFCHO2 exerts its inhibitory effect on the PI3K/AKT pathway by binding to DND1, we further performed a response experiment. The results indicated a significant reduction in the number of colonies formed in A375 and MV3 cells upon DND1 overexpression compared to the control group (Fig. 6A). The absorbance value at 450 nm was significantly reduced by DND1 overexpression compared to the control group (Fig. 6B). The number of A375 and MV3 cells penetrating the transwell compartment was significantly reduced by DND1 overexpression compared to the control group (Fig. 6C). The proportion of A375 and MV3 cell scratch area recovered was significantly reduced by DND1 overexpression compared to the control group (Fig. 6D). Furthermore, the expression of phosphorylated AKT (pAKT) decreased by DND1 overexpression compared to the control group, while the total AKT expression did not show significant changes (Fig. 6E). Thus, we can conclude that overexpression of DND1 can restore the promoting effect of overexpression of circFCHO2 on melanoma progression, and confirm that circFCHO2 can promote melanoma progression by combining with DND1 to remove the inhibition of PI3K/AKT signaling pathway (Fig. 7).

## Discussion

The previous work of the research group was to explore new key circular RNAs in melanoma and conduct functional research through bioinformatics database mining, which was relatively limited in scientificity and rigor. At present, although the sample preparation process is relatively convenient, the prefabricated chip method in circular RNA detection can only detect a limited number of circular RNAs compared to fresh samples. Therefore, we only use the prefabricated chip detection method as a verification method for the target circular RNA. In contrast, the application of next-generation sequencing technology with higher throughput and more accurate information can detect some novel circular RNAs in fresh tissue samples, which can further enhance the scientific and rigorous nature of research. In this study, the high-throughput next-generation sequencing technology was used to screen and analyze the differentially expressed circular RNAs in melanoma tissues of surgical patients in our hospital. To avoid possible interference from benign pigmentary disorders and to confirm the feasibility of the study, we compared the expression level of circFCHO2 in melanoma tissue and benign nevus tissue at the organizational level, and compared the expression level of circFCHO2 in melanoma cell lines and normal epidermal melanocytes at the cytological level. The results confirmed that circFCHO2 was only upregulated in melanin-related malignancies, thus ruling out possible interference from benign pigmentary disorders. As circFCHO2 has not yet been reported in the literature describing its related functions, we analyzed its clinical relevance based on the melanoma tissue microarray of our research center to confirm whether the study has more important clinical significance. Combined with the analysis of clinical information data from tissue microarray, we found that the overall survival and progression-free survival of melanoma patients with high circFCHO2 expression were lower than those with low circFCHO2 expression. The expression level of circFCHO2 exhibited associations with anatomical location, histological classification, lymph node metastasis, distal metastasis, and clinical staging. It serves as an independent risk factor for predicting the prognosis of melanoma patients. These findings underscore the close correlation of circFCHO2 with melanoma development, indicating its robust clinical relevance. Further exploration of its structure, function, and detailed mechanism of action is warranted. Whether circFCHO2 has the exact circular structure characteristics and stability of circular RNA is crucial for our subsequent related research. The results of RNase R treatment showed that circFCHO2 has a certain tolerance to RNase R, confirming the cyclization stability of circFCHO2. Using qRT-PCR to detect expression levels during RNase R treatment, we found that although mRNA expression levels decreased significantly, circRNA expression levels also decreased (while there was no statistically significant difference). We speculate that although circular RNA has relatively stable structural properties, it may still undergo partial degradation with storage time, environmental changes, temperature changes, external contact, and other conditions, leading to results similar to those presented in this experimental study.

With the gradual deepening of research on circular RNA in melanoma, its many functions and pathways have been gradually revealed, but there are still many functions of circular RNA that have not been fully defined (Mecozzi et al. 2021; Tang et al. 2021). Currently, approximately 80% of the reported biological functions of circular RNA are related to tumor progression or inhibition (Hsiao et al. 2017; Chen et al. 2018, 2017; Yang et al. 2017, 2019; Qiu et al. 2018; Zeng et al. 2018; Wang et al. 2018; Zhong et al. 2017; Abdelmohsen et al. 2017). Since tumor cells usually have a stronger ability to divide and proliferate than normal histiocytes, the ability to break through in situ to deep invasion, and the ability to migrate to other organs and tissues in various ways, we used various phenotypic experimental studies to investigate the changes in the proliferation, invasion, and migration ability of melanoma cells after knockdown and overexpression of the key circular RNA-circFCHO2 screened in the previous part of the study. Besides, the cell cycle is also an indicator of proliferation. The flow cytometry detection results show that the proportion of melanoma cells in each growth phase does not change significantly after overexpression of circFCHO2. However, the apoptosis detection results show that overexpression of circFCHO2 in melanoma cell lines can inhibit the apoptosis of melanoma cells to a certain extent. Therefore, we tentatively speculate that the growth-promoting effect of circFCHO2 on melanoma may achieve the ultimate proliferation effect by inhibiting cell apoptosis and indirectly increasing the number of cells in each phase of the cell cycle.

An increasing number of research reports indicate that circular RNA is intricately involved in the comprehensive regulation of tumorigenesis (including proliferation and apoptosis) and metastasis (encompassing invasion and migration). Furthermore, circular RNA is associated with various cellular processes such as the cell cycle, angiogenesis, metabolic reprogramming, aging, autophagy, stem cell characteristics, and chemical sensitivity (Hsiao et al. 2017; Chen et al. 2018, 2017; Yang et al. 2017, 2019; Qiu et al. 2018; Zeng et al. 2018; Wang et al. 2018; Zhong et al. 2017; Abdelmohsen et al. 2017; Li et al. 2020). Other regulatory effects mainly include cardiovascular and neurological function, inflammation and autoimmunity, wound repair and regeneration, and muscle production and adiposity, as well as unclear downstream effects (Liu and Chen 2022). Although these studies cover a wide range of areas, the specific laws of action they follow have not yet been summarized, indicating that there is still a significant gap between basic research and clinical practice. This highlights the necessity for further in-depth exploration in this field. Numerous studies have reported that circular RNA is implicated in the regulation of various signaling pathways associated with tumor progression (Hsiao et al. 2017; Chen et al. 2018, 2017; Yang et al. 2017, 2019; Qiu et al. 2018; Zeng et al. 2018; Wang et al. 2018; Zhong et al. 2017; Abdelmohsen et al. 2017), and melanoma progression is closely associated with multiple signaling pathways (Wei et al. 2020). However, previous studies have demonstrated that the regulatory effect of circular RNA on the pathway often requires one or more “intermediate” genes (Hsiao et al. 2017; Chen et al. 2018, 2017; Yang et al. 2017, 2019; Qiu et al. 2018; Zeng et al. 2018; Wang et al. 2018; Zhong et al. 2017; Abdelmohsen et al. 2017). Based on this, we further investigated the target genes of circFCHO2. The vast majority of studies on the function of circular RNA have reported that circular RNA acts as a miRNA “sponge” to regulate the function of ceRNA networks (Hsiao et al. 2017; Chen et al. 2018, 2017; Qiu et al. 2018; Zeng et al. 2018; Wang et al. 2018; Yang et al. 2019; Zhong et al. 2017), which is an overly simplistic and stereotypical impression of circular RNA. CDR1as was the earliest circular RNA to conduct functional research, which also led to the interaction network mechanism of ceRNA (Memczak et al. 2013; Hansen et al. 2013). A recent research report shows that CDR1as can interact with IGF2BP3 and inhibit its metastatic function (Hanniford et al. 2020). Another study discovered that CDR1as can interact with p53, inhibiting its separation from MDM2 (Thul et al. 2017). These research results inspire us that circular RNA may have multiple functional mechanisms, and previous studies have reported that circular RNA may have other modes of action besides ceRNA regulation mechanisms. We found differences in the secondary structure of circFCHO2 compared to most other circular RNA structures by searching it in the database. It has fewer miRNA binding sites, while it has more RNA binding protein binding sites. We therefore speculate that circFCHO2 may have a function of interacting with RNA binding proteins. Next, we pulled down all the protein molecules that can bind to circFCHO2 through the RNA pulldown experiment and performed protein mass spectrometry analysis of the pulled-down protein molecules. As a result, we found that the score of DND1 was the most significant, so we tentatively speculated that DND1 was the RNA binding protein most likely to bind to circFCHO2.

DND1 is a specific germ cell marker in vertebrates that belongs to the family of RNA-binding proteins (RBPs). Previous reports have shown that DND1 is critical for maintaining the survival and migration of primitive germ cells (Yamaji et al. 2017; Gross-Thebing and Raz 2020; Thul et al. 2017). Recent research reports have shown that DND1 is associated with some somatic cell tumors (Zhang and Godavarthi 2021). In liver cancer cells, studies have revealed that the overexpression of DND1 can suppress the characteristics of liver cancer stem cells, impede spheroid formation, restrain epithelial-mesenchymal transformation, and enhance the sensitivity of liver cancer cells to sorafenib therapy (Weiling et al. 2017). In breast cancer, patients exhibiting elevated levels of DND1 experience prolonged overall survival, and there exists a positive correlation between DND1 expression and the expression of the pro-apoptotic effector protein BIM in human breast cancer. Knockout of DND1 can reduce the expression of BIM and inhibit apoptosis of breast cancer cells (Cheng et al. 2017). In tongue squamous cell carcinoma cells, DND1 has been identified as a target for miR-24. The downregulation of DND1 by miR-24 results in reduced expression of the cyclin-dependent kinase inhibitor CDKN1B, a gene that is normally upregulated by DND1. This regulatory mechanism promotes cell proliferation (Liu et al. 2010). Several studies have unveiled and clarified the molecular mechanisms associated with the function of DND1 and its regulatory role in human malignant tumors. However, additional investigation is required to ascertain whether DND1 is specifically expressed aberrantly in these malignancies or induced by certain stress responses within tumor cells. Compared to other widely studied cancer-related genes such as KRAS, PTEN, and TP53, DND1 has a relatively short research history and its mechanism of action in different malignancies such as melanoma is still largely unknown. Therefore, in this research process, we conducted a more comprehensive exploration of the role and related regulatory mechanism of DND1, which was found to be associated with circular RNA in the progression of melanoma.

Since the correlation between DND1 and melanoma has not been reported yet, we first analyzed the clinical correlation between DND1 and melanoma patients through TCGA database bioinformatics. It indicated that melanoma patients with low DND1 expression levels had a significantly shorter overall survival period compared to those with high DND1 expression levels. Therefore, we speculate that DND1 may be a molecule that plays a significant inhibitory role in melanoma progression. Previous studies have demonstrated that circular RNA can block the function of another protein by binding to it, similar to a “binding” protein (Fang et al. 2018; Xueting et al. 2019; Bi et al. 2018; Zhu et al. 2020; Shen et al. 2020; Zhao et al. 2020; Lou et al. 2020). Based on the previous experimental results, we speculate that circFCHO2 also blocks the interaction between DND1 and signaling pathway proteins by this mechanism, thereby inhibiting its downregulation effect on the PI3K/AKT signaling pathway. Therefore, we also performed a response experiment. The results demonstrated that the overexpression of DND1 in melanoma cell lines had an inhibitory effect on the in vitro proliferation, invasion, and migration phenotype of melanoma cells, as well as on the activation of the PI3K/AKT signaling pathway induced by the overexpression of circFCHO2. This provisionally confirms our earlier speculation. The protein targets of the pathway blocked by the binding of circFCHO2 to DND1 still need to be identified and verified in further research. Based on the above series of experimental results, we can conclude that circFCHO2 in melanoma alleviates its inhibition of the PI3K/AKT signaling pathway by combining with DND1, and ultimately produces the biological effect of promoting the progression of melanoma.

## Conclusion

In summary, this study represents the initial identification of a novel circRNA, circFCHO2, characterized by overexpression in melanoma tissues. Elevated circFCHO2 levels were found to be associated with an unfavorable prognosis in melanoma patients. Our findings highlight the involvement of circFCHO2 in the regulation of the PI3K/AKT signaling pathway through its interaction with DND1, consequently fostering melanoma proliferation. Thus, our research sheds light on the oncogenic role of circFCHO2 in melanoma, suggesting its potential utility as both a biomarker and therapeutic target in the management of melanoma.

## Supplementary Information

Below is the link to the electronic supplementary material.

Supplementary file1 Additional file 1:Table S1. Sequences of siRNAs used for transient transfection. (DOCX 12 KB)

Supplementary file2 Additional file 2:Table S2. Sequences of primers used for qRT-PCR. (DOCX 12 KB)

Supplementary file3 Additional file 3:Table S3. List of primary antibodies used in the study. (DOCX 13 KB)

Supplementary file4 Additional file 4:Table S4. circFCHO2 FISH probe sequence. (DOCX 12 KB)

Supplementary file5 Additional file 5:Table S5. circFCHO2 RNA-pull down probe sequence. (DOCX 12 KB)

Supplementary file6 Additional file 6:Fig. S1. Relative circFCHO2 expression. A. The expression levels of 16 circRNA candidates were detected by qRT-PCR. B. qRT-PCR analysis of circFCHO2 level in benign nevus and melanoma tissues (n=9 for each group). C. qRT-PCR analysis of circFCHO2 level in different melanoma cell lines and PIG1, a normal epidermal melanocyte cell line. (TIF 6029 KB)

Supplementary file7 Additional file 7:Fig. S2. Effect of circFCHO2 on the cell cycle of melanoma cells. A. FCM was performed to assess the role of circFCHO2 in the cell cycle. CircFCHO2 was overexpressed in A375 cells compared with the vector group. (TIF 2023 KB)

Supplementary file8 Additional file 8:Fig. S3.Validation of DND1 knockdown and overexpression melanoma cell lines and signaling pathways. A-D. The efficacy of DND1 interference and overexpression was verified by qRT-PCR and western blotting. E-F. Western blot assay was used to detect the p-AKT and AKT levels in melanoma cells with different treatment, GAPDH was used as a negative control. (TIF 2023 KB)

Supplementary file9 Additional file 9: Supplementary Materials and Methods. (DOC 20 KB)

## Data Availability

Data and materials used and/or analyzed in this study are available from the corresponding authors on reasonable request.

## Declarations

### Approval of the research protocol by an Institutional Reviewer Board

The study was approved by the Independent Ethics Committee of Zhongshan Hospital, Fudan University.

### Registry and the registration no. of the study

Y2020-220.

### Animal studies

Animal studies were approved by the Animal Ethics Committee of Zhongshan Hospital, Fudan University (2020–140).
