# Supplementary material for: Circular RNA circFCHO2(hsa_circ_0002490) promotes the proliferation of melanoma by directly binding to DND1
Source: Cell Biol Toxicol. 2024 Feb 5;40(1):9. doi: 10.1007/s10565-024-09851-y (PMC10838848; doi:10.1007/s10565-024-09851-y)
Supplement: Supplementary file 1 — Supplementary file1 Additional file 1:Table S1. Sequences of siRNAs used for transient transfection. (DOCX 12 KB) [file 10565_2024_9851_MOESM1_ESM.docx]

Supplementary table 1:

**Legend:Table S1. Sequences of siRNAs used for transient transfection.**

| **circFCHO2-si1** |  |
| --- | --- |
| Forward | 5’-GCACAGCUUGUGUUCAGGUGUTT-3’ |
| Reverse | 5’-ACACCUGAACACAAGCUGUGCTT-3’ |
| **circFCHO2-si2** |  |
| Forward | 5’-ACAGCUUGUGUUCAGGUGUGUTT-3’ |
| Reverse | 5’-ACACACCUGAACACAAGCUGUTT-3’ |
| **DND1-si1** |  |
| Forward | 5’-GUGUACGAGCACCAGCUUAUCdTdT-3’ |
| Reverse | 5’-GAUAAGCUGGUGCUCGUACACdTdT-3’ |
| **DND1-si2** |  |
| Forward | 5’-GGGUGAAUCCAGAGAACAAGGdTdT-3’ |
| Reverse | 5’-CCUUGUUCUCUGGAUUCACCCdTdT-3’ |
