# Supplementary material for: Circular RNA circFCHO2(hsa_circ_0002490) promotes the proliferation of melanoma by directly binding to DND1
Source: Cell Biol Toxicol. 2024 Feb 5;40(1):9. doi: 10.1007/s10565-024-09851-y (PMC10838848; doi:10.1007/s10565-024-09851-y)
Supplement: Supplementary file 2 — Supplementary file2 Additional file 2:Table S2. Sequences of primers used for qRT-PCR. (DOCX 12 KB) [file 10565_2024_9851_MOESM2_ESM.docx]

Supplementary table 2:

**Legend:Table S2. Sequences of primers used for qRT-PCR.**

| **circFCHO2** |  |
| --- | --- |
| Forward | 5’-TTCTTCCAAATGCACAGCTTGTG-3’ |
| Reverse | 5’-AATACACTTGGTGGGATCTGCTC-3’ |
| **FCHO2** |  |
| Forward | 5’-GTCGACATGGTCATGGCGCAT TTCGTGGAGA-3’ |
| Reverse | 5’-GTCGACTCAACAATCTGCCAAGTATCGTCCAGT-3’ |
| **U6** |  |
| Forward | 5’-CTCGCTTCGGCAGCACA-3’ |
| Reverse | 5’-AACGCTTCACGAATTTGCGT-3’ |
| **GAPDH** |  |
| Forward | 5’-GGTATGACAACGAATTTGGC-3’ |
| Reverse | 5’-GAGCACAGGGTACTTTATTG-3’ |
