# Supplementary material for: Circular RNA circFCHO2(hsa_circ_0002490) promotes the proliferation of melanoma by directly binding to DND1
Source: Cell Biol Toxicol. 2024 Feb 5;40(1):9. doi: 10.1007/s10565-024-09851-y (PMC10838848; doi:10.1007/s10565-024-09851-y)
Supplement: Supplementary file 3 — Supplementary file3 Additional file 3:Table S3. List of primary antibodies used in the study. (DOCX 13 KB) [file 10565_2024_9851_MOESM3_ESM.docx]

Supplementary table 3:

**Legend:Table S3. List of primary antibodies used in the study.**

| Antibody | Applications | Company |
| --- | --- | --- |
| Ki-67 | WB, IHC, IF | Abcam (ab16667) |
| GAPDH | WB, IF | Abcam (ab8245) |
| DND1 | WB, IF, IHC, IP | Santa Cruz (sc-130493) |
| AKT | WB, F, IF, IHC, IP, IHC | Abcam (ab8805) |
| p-AKT | WB, IP, IF, IHC | Abcam (ab38449) |
| IgG | WB, IHC, IP, CHIP, F | Abcam (ab172730) |

**Abbreviations:** WB, western blot; IHC, immunohistochemistry; IF, immunofluorescence; IP, immunoprecipitation; F, flow cytometric analysis; RIP, RNA immunoprecipitation; CHIP, Chromatin Immunoprecipitation.
