# Supplementary material for: Circular RNA circFCHO2(hsa_circ_0002490) promotes the proliferation of melanoma by directly binding to DND1
Source: Cell Biol Toxicol. 2024 Feb 5;40(1):9. doi: 10.1007/s10565-024-09851-y (PMC10838848; doi:10.1007/s10565-024-09851-y)
Supplement: Supplementary file 4 — Supplementary file4 Additional file 4:Table S4. circFCHO2 FISH probe sequence. (DOCX 12 KB) [file 10565_2024_9851_MOESM4_ESM.docx]

Supplementary table 4:

**Legend:Table S4. circFCHO2 FISH probe sequence.**

| Name | Sequence |
| --- | --- |
| circFCHO2 FISH probe | 5’-ACCCCGTGACACACCTGAACACAA-3’ |
