# Supplementary material for: Circular RNA circFCHO2(hsa_circ_0002490) promotes the proliferation of melanoma by directly binding to DND1
Source: Cell Biol Toxicol. 2024 Feb 5;40(1):9. doi: 10.1007/s10565-024-09851-y (PMC10838848; doi:10.1007/s10565-024-09851-y)
Supplement: Supplementary file 5 — Supplementary file5 Additional file 5:Table S5. circFCHO2 RNA-pull down probe sequence. (DOCX 12 KB) [file 10565_2024_9851_MOESM5_ESM.docx]

Supplementary table 5:

**Legend:Table S5. circFCHO2 RNA-pulldown probe sequence.**

| Name | Sequence |
| --- | --- |
| circFCHO2 RNA-pulldown probe-Positive | 5’-GACCCCGTGACACACCTGAACACA-3’ |
| circFCHO2 RNA-pulldown probe-Negative | 5’-GCTTGTGTTCAGGTGTGTCACGGG-3’ |
