# Supplementary figures and images for: Circular RNA circFCHO2(hsa_circ_0002490) promotes the proliferation of melanoma by directly binding to DND1
Source: Cell Biol Toxicol. 2024 Feb 5;40(1):9. doi: 10.1007/s10565-024-09851-y (PMC10838848; doi:10.1007/s10565-024-09851-y)

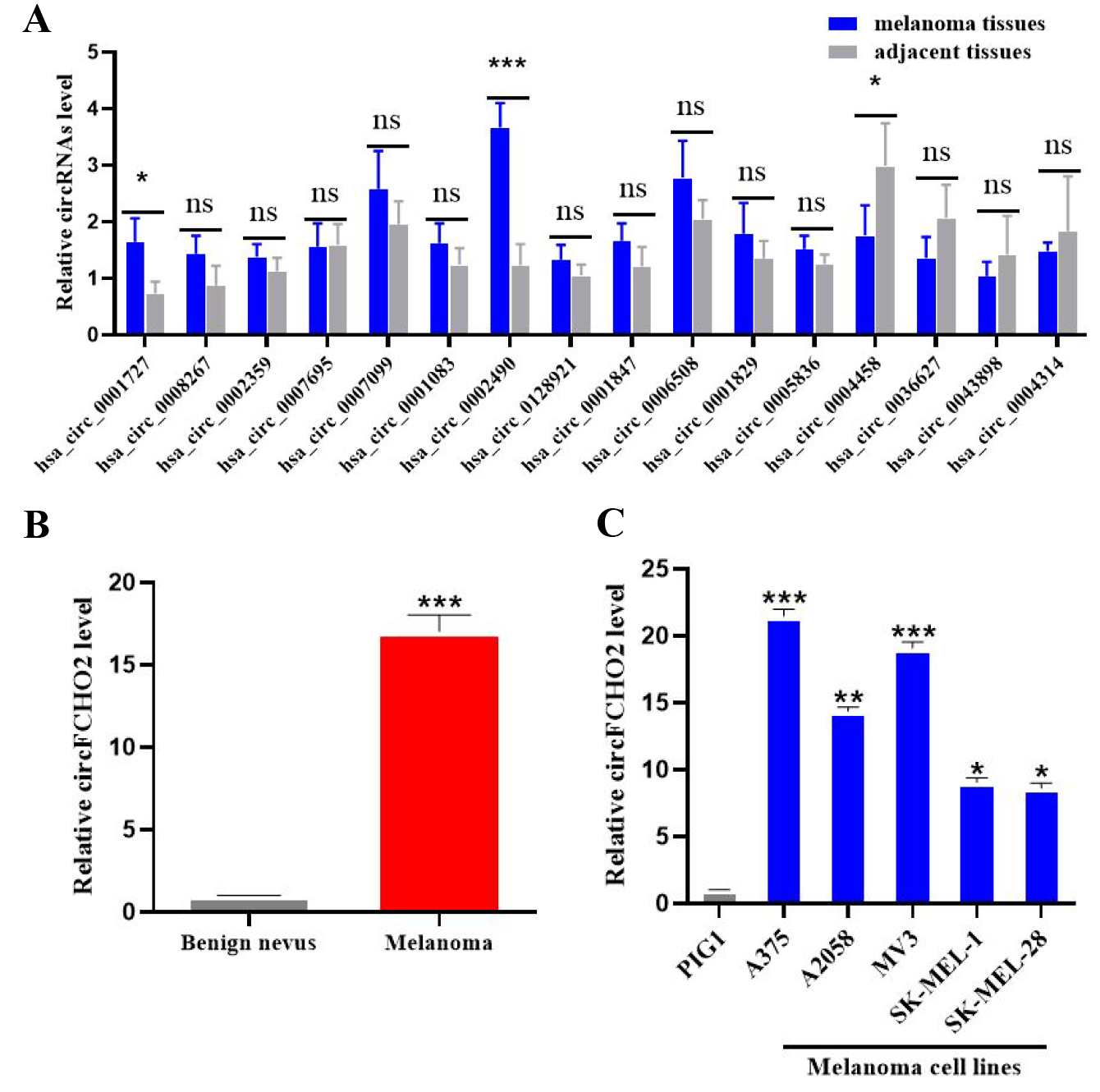

Supplement: Supplementary file 6 — Supplementary file6 Additional file 6:Fig. S1. Relative circFCHO2 expression. A. The expression levels of 16 circRNA candidates were detected by qRT-PCR. B. qRT-PCR analysis of circFCHO2 level in benign nevus and melanoma tissues (n=9 for each group). C. qRT-PCR analysis of circFCHO2 level in different melanoma cell lines and PIG1, a normal epidermal melanocyte cell line. (TIF 6029 KB) [file 10565_2024_9851_MOESM6_ESM.tif]

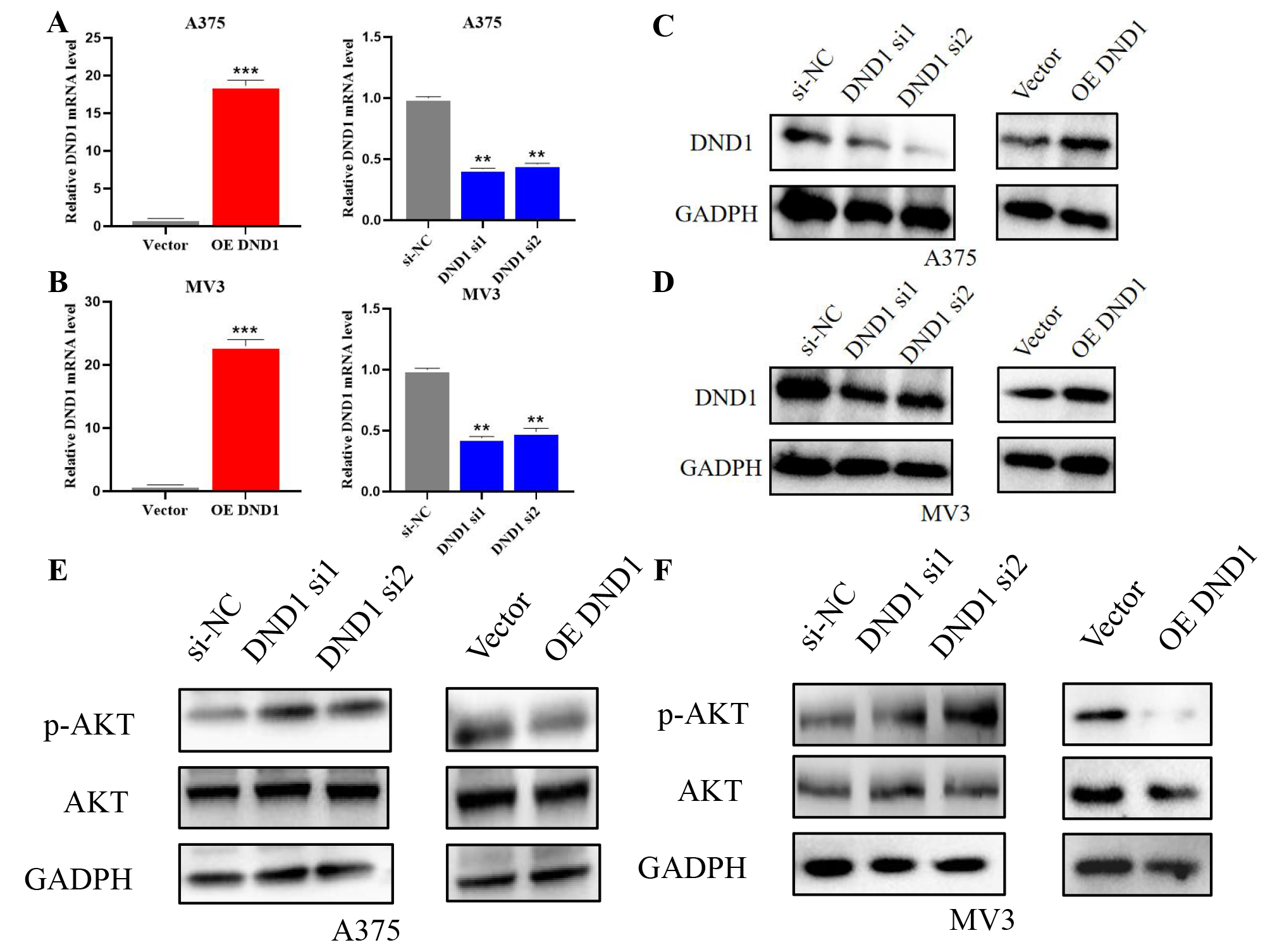

Supplement: Supplementary file 7 — Supplementary file7 Additional file 7:Fig. S2. Effect of circFCHO2 on the cell cycle of melanoma cells. A. FCM was performed to assess the role of circFCHO2 in the cell cycle. CircFCHO2 was overexpressed in A375 cells compared with the vector group. (TIF 2023 KB) [file 10565_2024_9851_MOESM7_ESM.tif]
